# Cross Fostering Experiments Suggest That Mice Songs Are Innate

**DOI:** 10.1371/journal.pone.0017721

**Published:** 2011-03-09

**Authors:** Takefumi Kikusui, Kaori Nakanishi, Ryoko Nakagawa, Miho Nagasawa, Kazutaka Mogi, Kazuo Okanoya

**Affiliations:** 1 Companion Animal Research, School of Veterinary Medicine, Azabu University, Sagamihara, Kanagawa, Japan; 2 Laboratory for Biolinguistics, Mind and Intelligence Research Core, Brain Science Institute (BSI), RIKEN, Wako, Saitama, Japan; 3 ERATO, Okanoya Emotional Information Project, Japan Science and Technology Corporation, Wako, Saitama, Japan; Freie Universitaet Berlin, Germany

## Abstract

**Background:**

Vocal learning is a central functional constituent of human speech, and recent studies showing that adult male mice emit ultrasonic sound sequences characterized as “songs” have suggested that the ultrasonic courtship sounds of mice provide a mammalian model of vocal learning.

**Objectives:**

We tested whether mouse songs are learned, by examining the relative role of rearing environment in a cross-fostering experiment.

**Methods and Findings:**

We found that C57BL/6 and BALB/c males emit a clearly different pattern of songs with different frequency and syllable compositions; C57BL/6 males showed a higher peak frequency of syllables, shorter intervals between syllables, and more upward frequency modulations with jumps, whereas BALB/c males produced more “chevron” and “harmonics” syllables. To establish the degree of environmental influences in mouse song development, sons of these two strains were cross-fostered to another strain of parents. Songs were recorded when these cross-fostered pups were fully developed and their songs were compared with those of male mice reared by the genetic parents. The cross-fostered animals sang songs with acoustic characteristics - including syllable interval, peak frequency, and modulation patterns - similar to those of their genetic parents. In addition their song elements retained sequential characteristics similar to those of their genetic parents' songs.

**Conclusion:**

These results do not support the hypothesis that mouse “song” is learned; we found no evidence for vocal learning of any sort under the conditions of this experiment. Our observation that the strain-specific character of the song profile persisted even after changing the developmental auditory environment suggests that the structure of these courtship sound sequences is under strong genetic control. Thus, the usefulness of mouse “song” as a model of mammalian vocal learning is limited, but mouse song has the potential to be an indispensable model to study genetic mechanisms for vocal patterning and behavioral sequences.

## Introduction

Many animals, including humans, use vocal signals to communicate with conspecifics. Song is a long, complex vocalization of several acoustic elements arranged in specific sequences [1]. [2]. While most mammals, birds [3], and frogs [4] tested show only genetically regulated patterns of vocalizations, several rare groups of birds (songbirds, parrots, hummingbirds) and mammals (whales, bats and humans) also learn vocalizations. They learn them through social imitation, with different degrees of innate constraints depending on the species [5], [6]. In most species, vocal learning occurs mainly during juvenile development. In zebra finches, for instance, approximately 30 days after hatching, young males start producing unstructured sounds. The onset of vocal learning after exposure to a song model from a tutor, usually the father, is marked by the rapid emergence of structured sounds. To learn a song, the bird has to compare these sounds with a memory template of the song model using auditory feedback [7]. Learning songs is achieved by transforming and differentiating prototype sounds until they resemble the different syllables of the song model. This type of vocal learning for which neural and molecular substrates have been well documented [3] is similar to human spoken language learning [7].

The mouse, *Mus musculus*, is a genetically and neurochemically well-described mammalian organism. Mice emit ultrasonic vocalizations with frequencies higher than 30 kHz, which is far beyond the human audible range [8]. Mice produce ultrasonic vocalizations in 2 social contexts: first, pups' production of ‘‘isolation calls’’ in cold conditions or when they are separated from the dam [9], [10]; second, males emitting ‘‘ultrasonic vocalizations’’ in the presence of females or when they are stimulated by the female's urinary pheromones [11]. Recent studies have demonstrated that ultrasonic song vocalizations of male mice have behavioral features similar to those of bird songs, including discrete syllables with temporal sequencing, repeated phrases, and variability among individuals [12].

The B6D2F1strain of male mice showed individual differences in syllable usage and the temporal structure of their songs as reported by Holy and Guo [12]. Furthermore, mating has been shown to change the quality and quantity of male ultrasonic vocalization [13]. These findings lead to the hypothesis that male mouse songs may have an experience-dependent phenotype. However, the influence of social environments during the early developmental period, in which songbirds learn the prototype of songs from their tutors as clearly shown by cross-fostering studies [14], has not been examined.

To elucidate genetic and environmental effects on mouse songs, we conducted a cross-fostering study to understand the effects of the social experience during the juvenile developmental period on song development. First, we compared 2 strains of inbred C57BL/6 and BALB/c males and found that these 2 strains of male mice emitted a different pattern of songs with regard to frequency, inter-syllable intervals, and syllable composition. C57BL/6 males showed a higher peak frequency of syllables and more frequency-modulated syllables with 1 or multiple jumps and short- and upward syllables, whereas BALB/c males produced more chevron-, flat-, and harmonics-syllables. None of these strain-specific parameters were affected by cross-fostering. Therefore, developmental social environments appear to have no significant role in adult male songs of mice. In other words, mouse songs do not seem to involve imitative learning.

## Results

### Strain differences in ultrasonic songs

#### Song parameters

**Figure 1:**
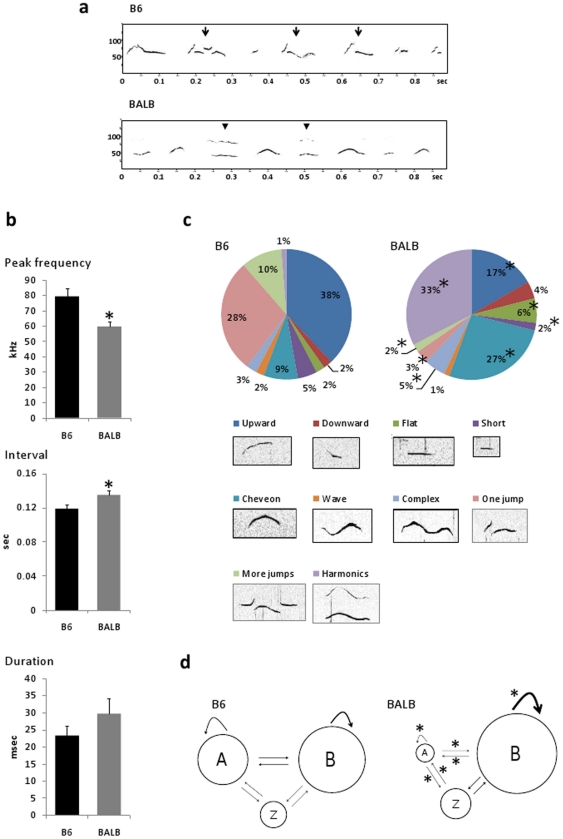
Strain-specific characteristics of male mice songs. (a) Sound spectrograms of ultrasonic songs in B6 (upper) and BALB (lower) male mice. B6 males showed a higher peak frequency of syllables ranging from 70–110 kHz, shorter intervals between syllables, and more upward frequency modulations with jumps (arrows), whereas BALB males produced more “chevron” and “harmonics” syllables (arrow head). (b) The mean syllable peak frequency and inter-syllable interval significantly differed between B6 and BALB mice, but syllable duration was not. Data are expressed as mean ± SEM; *p<0.05 between strains. (c) Pie graphs showing percentages of the 10 categories of song syllables in B6 and BALB mice. Percentages were calculated in each strain as the number of syllables in each category for each subject/total number of syllables analyzed in each subject. The number of total syllables analyzed was: 6179 for B6 mice and 6244 for BALB mice. B6 mice produced more “short,” “one jump,” and “more jumps” syllables than BALB mice, whereas BALB mice produced more “flat”, “chevron”, “complex”, and “harmonics” syllables; *p<0.05 between strains. (d) In the sequential analysis, we divided all syllable types into 2 categories, namely, A (syllables with frequency jumps) and B (syllables without jumps). Z indicates silent gaps longer than 0.25 s. Circles represent the percentage of syllable types, and the thickness of the arrows represents the transition probabilities. The sequential analyses of syllables demonstrated strain-specific patterns; B6 mice showed more transition from A to A, A to B, A to Z, B to A, and Z to A than BALB mice and BALB mice showed more B to B self transition compared to that in B6 mice; *p<0.05 between strains.

When a male subject encountered a female, he emitted complex ultrasounds. Sound spectrograms demonstrated that B6 males showed a peak at 70–80 kHz, and BALB males at 50–60 kHz (Fig. 1a and Audio S1 and S2). The comparison between B6 and BALB mice revealed that the average peak frequency of syllables was lower in BALB males (Mann–Whitney test, p<0.005), the average interval between syllables was longer in BALB males (Mann–Whitney test, p<0.005), but the number and duration of syllables (Fig. 1b) emitted in the 3-min test did not differ significantly (B6, 240±45 times/min; BALB, 257±32 times/min).

#### Syllable category analysis

According to previous studies [15], each syllable was identified as 1 of 10 distinct categories: “upward,” “flat,” “chevron,” “complex,” “more jumps,” “downwards,” “short,” “wave,” “one jump,” or “harmonics”. The resulting pie graph indicated strain differences in the distribution of syllable categories (Fig. 1c). MANOVA, with the strain as the main factor and the probabilities of each syllable occurrence (10 in total) as dependent variables, revealed a significant between-group difference (F(9,3) = 69.7, p<0.0001). A post hoc t-test showed strain differences in 8 of the 10 syllable categories (Fig. 1c). B6 mice produced more “upward,” “short,” “one jump,” and “more jumps” syllables than BALB/c mice (p<0.05, t-test). In contrast, BALB/c mice produced more “flat,” “chevron,” “complex,” and “harmonics” syllables (p<0.05, t-test).

#### Sequential analyses of syllables

The sequential patterns of B6 and BALB mice songs are shown in Fig. S2. All 10 syllable categories were included in the analysis. Because these patterns were overly complicated for rigorous analysis, the syllable categories were lumped into 2 large types, namely, syllables with jumps (A) and other syllable types (B). The gap (more than 0.25 s) between each syllable bout is represented by Z. B6 and BALB mice showed distinct transitional patterns of the song syllables. MANOVA, with the strain as the main factor and the probabilities of each syllable transition (8 in total) as dependent variables, revealed a significant strain difference in the transition patterns (F(7,5) = 4.92, p<0.05). Post hoc t-tests showed a greater occurrence of transitions from types A to A, A to B, B to A, A to Z, and Z to A in B6 than in BALB mice, whereas BALB mice showed more B to B self-transition compared to B6 mice (Fig. 1d).

### Comparison between the fostered groups and naturally-reared sons

#### Sonograms

**Figure 2:**
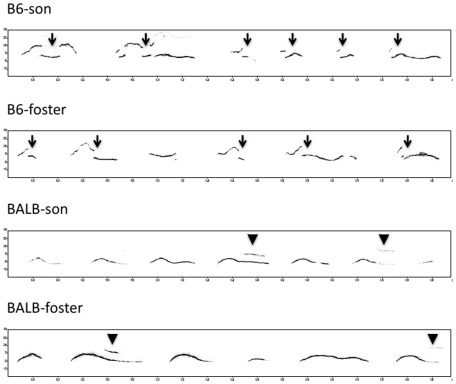
Sonograms of ultrasonic songs in fostered males. Sonograms of ultrasonic songs recorded from B6-son, B6-foster, BALB-son, and BALB-foster male mice. Cross-fostered mice showed similar patterns to those of normally reared mice, and the effects of the rearing environment were not obvious. B6-son and B6-foster mice showed a higher peak frequency of syllables, shorter intervals between syllables, and more upward frequency modulations with jumps (arrows), whereas BALB-son and BALB-foster males produced more “chevron” and “harmonics” syllables (arrow head).

Sound spectrograms demonstrated that B6-sons and B6-foster males showed a peak at 70–80 kHz, whereas BALB mice showed a peak at 50–60 kHz (Fig. 2 and Audio S3, S4, S5 and S6).

#### Song parameters

**Figure 3:**
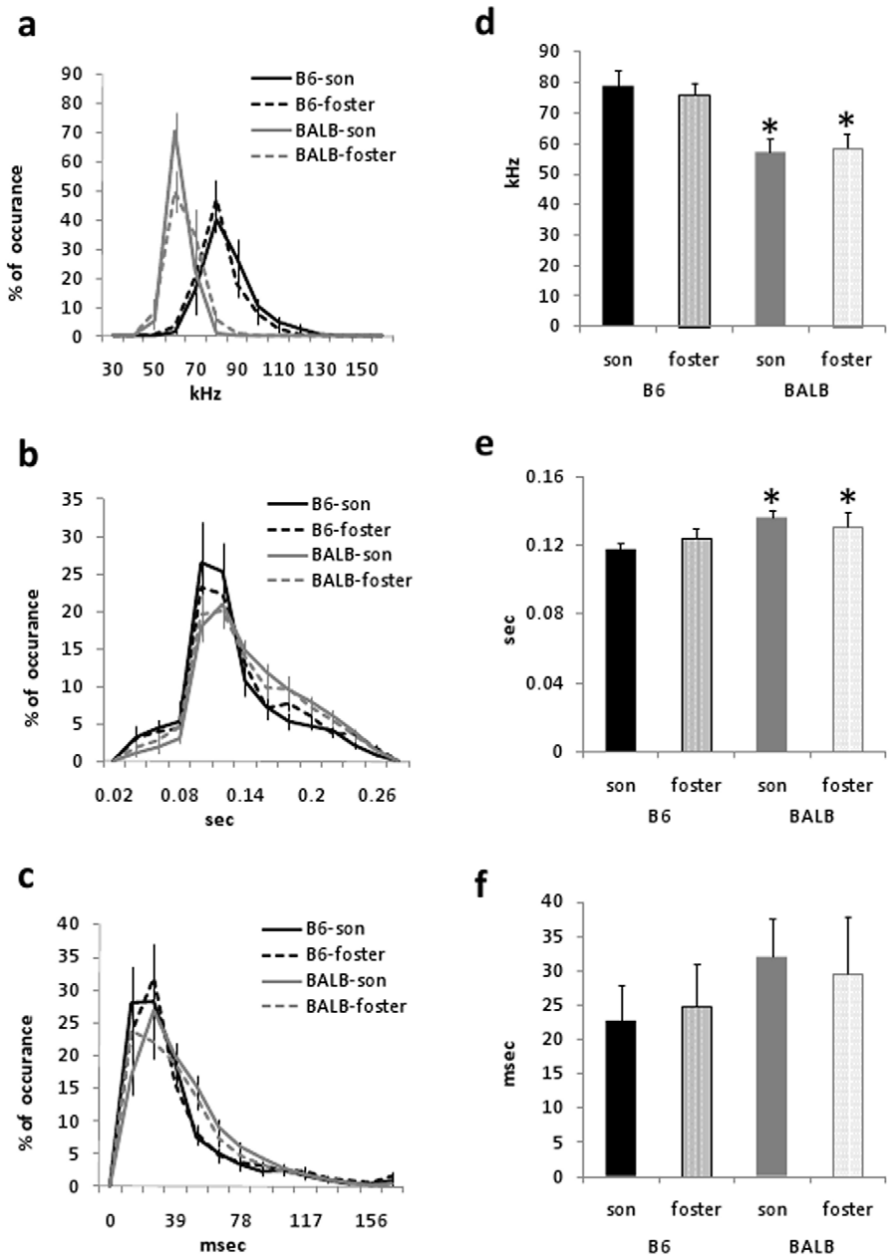
Song parameters of fostered males. Song parameters in B6-son, B6-foster, BALB-son, and BALB-foster male mice. The distribution histogram of the peak frequency (a) and intervals (b), but not the duration (c), of the syllables demonstrated significant strain differences, regardless of the fostering. Mean peak frequency (d) and interval (e) significantly differed between genetic B6 and BALB groups. Data are expressed as mean ± SEM; *p<0.05 vs. B6-son and B6-foster mice.

We compared songs between fostered groups, and found that the main strain differences we quantified were not affected by fostering. BALB cross-fostered males still showed a lower peak frequency (F(1,20) = 106.5, p<0.0001) and longer inter-syllable intervals (F(1,20) = 9.67, p<0.01) than B6-fostered males (Fig. 3). The syllable duration and the number of syllables emitted in the 3-min test were equivalent in all groups (B6-son, 225±56 times/min; B6-foster, 242±45 times/min; BALB-son, 225±33 times/min; BALB-foster, 249±37 times/min).

#### Syllable category analysis

**Figure 4:**
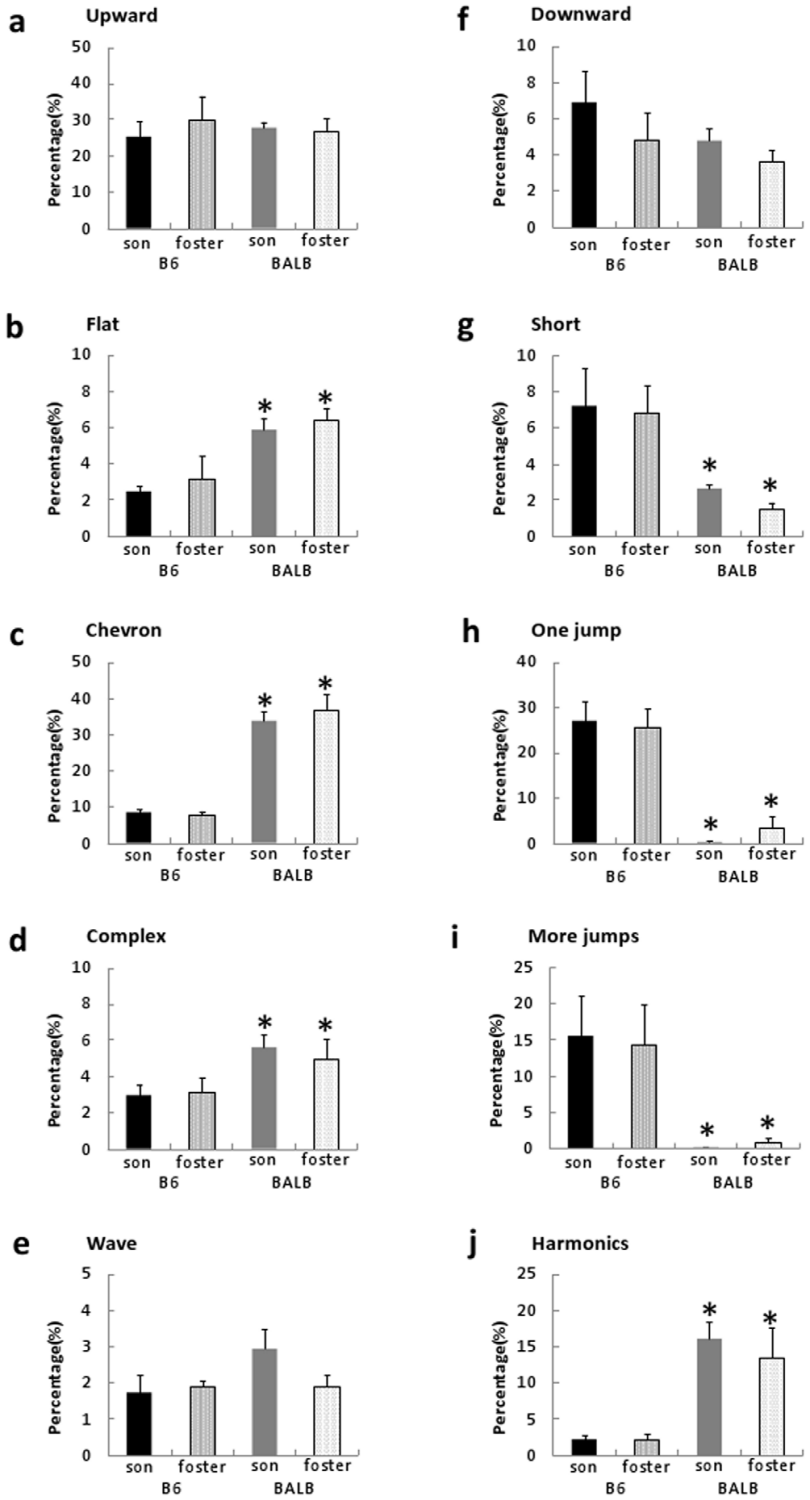
Appearance ratio of the song syllables in fostered males. The appearance ratio of each of the 10 syllable categories in B6-son, B6-foster, BALB-son, and BALB-foster mice. Genetic B6 groups produced more “short,” “one jump,” and “more jumps” syllables than BALB/c mice, whereas genetic BALB groups produced more “flat,” “chevron,” “complex,” and “harmonics” syllables. Data are expressed as mean ± SEM; *p<0.05 vs B6-son and B6-foster mice.

**Figure 5:**
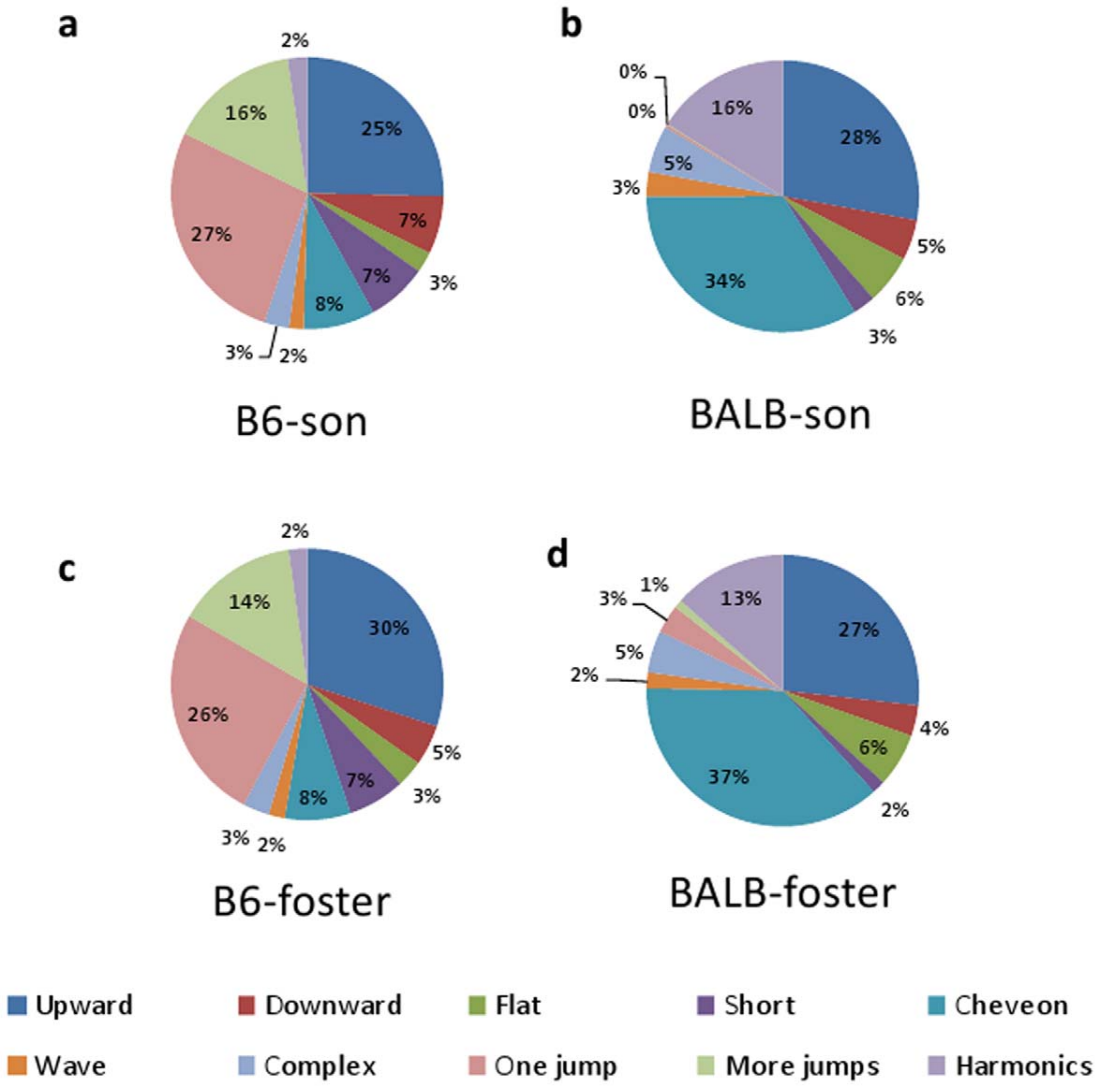
Distribution pattern of the song syllables in fostered males. Pie graphs showing the percentages of the 10 categories of song syllables in B6-son (a), BALB-son (b), B6-foster (c), and BALB-foster (d) mice. Percentages were calculated in each strain as the number of syllables in each category for each subject/total number of syllables analyzed in each subject. The total syllables determined are as follows: 5487 syllables; B6-son; 6414 syllables, B6-foster; 4973 syllables, BALB-son; 6963 syllables, BALB-foster.

MANOVA revealed a significant effect of strain (F(9,9) = 25.9, p<0.0001), but not of fostering (F(9,9) = 0.91, p = 0.55) or an interaction of strain and fostering (F(9,9) = 0.41, p = 0.89). Regardless of fostering experience, B6 mice produced more “short,” “one jump,” and “more jumps” syllables than BALB mice (Fig. 4, p<0.05). In contrast, BALB mice produced more “flat,” “chevron,” “complex,” and “harmonics” syllables (Fig. 4, p<0.05). The proportions of syllables within each category are shown in Fig. 5 which indicates that the differences in the appearance of syllable categories were mainly dependent on the strain of the mice.

#### Sequential analyses of syllables

**Figure 6:**
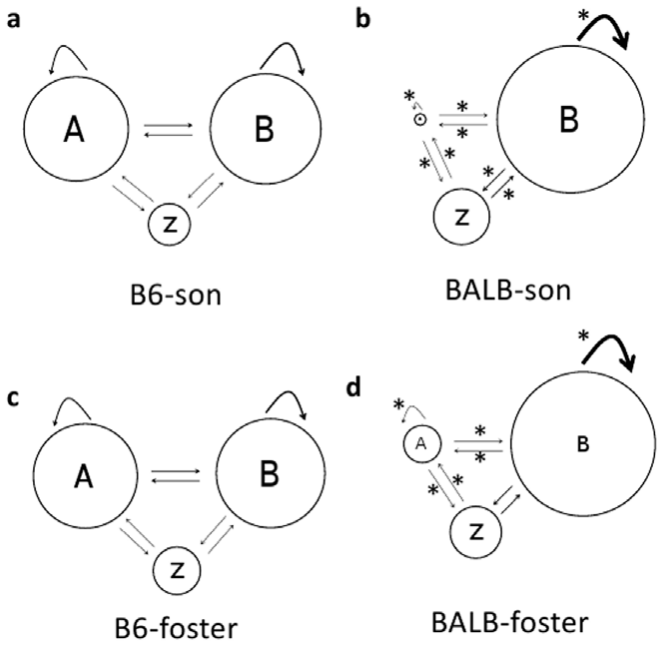
Sequential analysis of syllables in fostered males. Sequential analyses of syllables demonstrated strain-specific patterns; BALB-son mice showed a greater occurrence of B to B self transitions, and B to Z and Z to B transitions, as well as a lower occurrence of A to A self-transitions and A to B, B to A, A to Z, and Z to A transitions compared to B6-son and B6-foster mice. BALB-foster mice demonstrated greater occurrence of type B to B self-transitions and a lower occurrence of A to A, A to B, B to A, A to Z, and Z to A transitions compared to B6-son and B6-foster mice. Circles represent the percentage of syllable types, and the thickness of the arrows represents the transition probabilities; *p<0.05 vs. B6-son and B6-foster mice.

Regardless of fostering, B6 and BALB mice showed distinct transitional patterns of the song syllables, and these characteristics were displayed by cross-fostered males. MANOVA revealed a strain difference (F(6,12) = 24.6, p<0.0001), but no fostering effect (F(6,12) = 0.655, p = 0.687) and no interaction between these (F(6,12) = 1.56, p = 0.241). A Bonferonni post hoc test revealed that sons of BALB mice showed a greater occurrence of B to B self-transitions, B to Z and Z to B transitions as well as a lower occurrence of A to A self-transitions, A to B, B to A, A to Z, and Z to A transitions compared to sons of B6 and B6-foster male mice (p<0.05, Fig. 6). BALB-foster mice demonstrated a greater occurrence of B to B self-transitions and a lower occurrence of A to A, A to B, B to A, A to Z, and Z to A transitions compared to sons of B6 and B6-foster mice (p<0.05, Fig. 6).

## Discussion

In the present study, we revealed that B6 and BALB male mice showed distinct patterns and sound profiles of songs when encountering a female. Our syllable categories are similar to those reported in earlier studies [12], [15]–[17]. The average peak frequency of syllables was higher in B6 mice, and the average interval between syllables was shorter in B6 mice. In addition, B6 mice produced more “upward,” “short,” “one jump,” and “more jumps” syllables than BALB mice; whereas BALB mice produced more “flat,” “chevron,” “complex,” and “harmonics” syllables. By using the cross-fostering procedure, we further showed that these strain differences remained even after the pups were cross-fostered to another strain of parents, suggesting that the strain-specific song profile is determined by genetic factors and is independent of the juvenile social auditory environment.

Studies of the natural history of mice have demonstrated that a pair of male and female mice lives in a nest together with their juveniles [18]. In the laboratory the female goes into estrus around the time of delivery as indicated by an increase in estrogen levels, the so-called postpartum estrus [19], [20]. The odor of female urine stimulates the males to sing [12]; therefore, the pups can be exposed to male songs especially when the mother comes into the round of the reproductive cycle. Since the mouse can hear from at least postnatal day 10 [21], pups in the juvenile period have sufficient opportunity to be exposed to adult male songs. To be sure, we recorded in the laboratory one pair of B6 and one pair of BALB male and female continuously every day for three weeks after pup delivery, and found that the pair generated over 200 seconds of vocalizations each day, and these were always during the dark period (unpublished data). Thus, although not directly measured, we believe that the male mice in the cross fostering studies sang during the cross-fostering period. In this study, the fostered mice were housed in mixed strains of the same age in the post-weaning period, to standardize the possibility of hearing songs from littermates. If the strain differences reported in Fig. 1 had been the result of learning, our methods would have ensured that this rearing condition was sufficient to establish such strain differences. In fact, this is a general breeding condition utilized by most laboratories [22]. Therefore, our procedures should have been able to detect the effect of cross-fostering rearing environments, but we did not observe any of such effects.

Several studies have demonstrated that female mice show attraction to male songs [23], [24]. In these studies, however, a 2-choice test presenting 2 types of songs was not conducted; therefore, it remains a question whether female mice have a preference for a specific character of songs, as shown in songbirds [25]. Furthermore, female mice have been shown to respond to synthetic 70 kHz ultrasounds presented behind a devocalized male mouse [24] and to pup vocalizations [9], in which the observed syllable categories are similar to adult male songs [26]. In a recent study, female mice were shown to be able to distinguish between a familiar male song and an unfamiliar one based on the social experience of a short-term encounter and showed investigative behavior toward the unfamiliar song, implying that female mice can distinguish the individual profile of the songs [27]. These results suggest that a certain level of ultrasound complexity is sufficient to attract female mice, although the value of learning songs for male mice to achieve reproductive success remains unclear.

Recent studies have demonstrated that ultrasonic vocalization of mouse pups is affected by genes related to neuropsychiatric disorders such as Autism [15], [28]. These genetic approaches could reveal the genes that regulate ultrasonic vocalization in mice. For example, the function of Foxp2, a transcription factor shown to be related to a human language disorder [29], is involved in pup isolation calls. When human-type FoxP2 was inserted into the mice genome, isolation call pitch increased [30]. In addition, when FoxP2 was knocked out [31] or a FoxP2 mutation corresponding to the human language disorder was knocked in [32], the number of isolation calls decreased. However, these transgenic mice were tested with maternal separation-induced pup ultrasound vocalizations, not with male courtship songs. Therefore, it is of interest to test whether these genetically modified mice would show the quantitative and qualitative differences in adult male songs we observed. The complexity of the song pattern itself raises an interest in understanding the neural and molecular mechanisms controlling song in mammals.

Here we showed that imitative vocal learning is not involved in the strain specificity of mouse songs. Vocal learning requires two independent processes. First, the animal must have voluntary control over the vocal output. Second, the animal should be able to match its vocal output with the externally acquired auditory memory. For the first process, the existence of the direct motor pathway connecting the oro-facial motor cortex and the medullar phonatory and respiratory areas, including the nucleus ambiguus, has been suggested as an anatomical substrate responsible for vocal plasticity [3]. In fact, this cortico-bulber pathway for vocal plasticity exists in humans but not in non-human primates [33]. This pathway is also found in oscine songbirds such as the zebra finch and the canary but not in pigeons [34]. Since humans and oscine songbirds are vocal learners and non-human primates and pigeons are vocal non-learners, the existence of this pathway coincides with vocal learning.

Arriaga et al. reported singing-related gene expression in mice cingulated, motor cortex and basal ganglia [35]. They also reported the existence of the cortico-bulber pathway for vocal plasticity in mice [36], which should be related to the observed vocal complexity of the mouse song. Our data, which show no effect of the auditory environment by tutors on mouse song, may appear contradictory to these findings. However, vocal plasticity alone does not guarantee vocal learning, since vocal-auditory matching is also required for vocal learning to occur. A certain degree of voluntary vocal plasticity may be necessary in animals with complex vocalizations to maintain a stable performance even without learning. It may be interesting to examine the anatomical pathways in animals with complex vocalizations but without learning abilities, including sub-oscines [37] and gibbons [38]. Further, even though there was no clear evidence of vocal learning in the mice examined in this study, there may be other factors that modulate the phonetic and sequential variability of male songs. It is often assumed that highly variable songs are suggestive evidence of vocal learning. As seen from our sonograms and sequence analyses, mice songs are highly variable yet we find evidence that they are innate. This variability could be generated by a random pattern generator independent of learning or by some hormonal influence [39]. In either case, our findings indicate that the presence of variability does not automatically mean the presence of vocal learning.

### Conclusion

Our results show that the auditory environment does not affect song phenotypes in mice, and, thus, vocal learning does not appear to be involved in mouse songs. Nevertheless, mouse song is a very complex behavior, with at least 10 categories of vocal tokens and complex note-to-note transition rules. Even if this phenotype is largely controlled by genetic factors and only limited learning is involved, we can still pose interesting questions regarding the genetic encoding of acoustic categories and the neural mechanisms involved in sequence generation. Thus, the mouse song should remain an important model in which to study the biological basis of complex communicative behavior, including spoken human language.

## Materials and Methods

### Animals

BALB/cAJcl (BALB) and C57BL/6JJcl (B6) mice were originally obtained from Japan Clea Co. Ltd. (Japan Clea, Yokohama, Japan) and bred in our laboratory. Food and water were given ad libitum, and all the animals were kept at a constant temperature (23±1°C) and humidity (40%±10%) under a 12-h light:dark cycle (light on at 0600). All experiments were conducted in accordance with the guideline of the "Policies Governing The Use of Live Vertebrate Animals" by Azabu University, and were approved by The Ethical Committee for Vertebrate Experiments (ID# 070418).

### Pairing and cross-fostering

**Figure 7:**
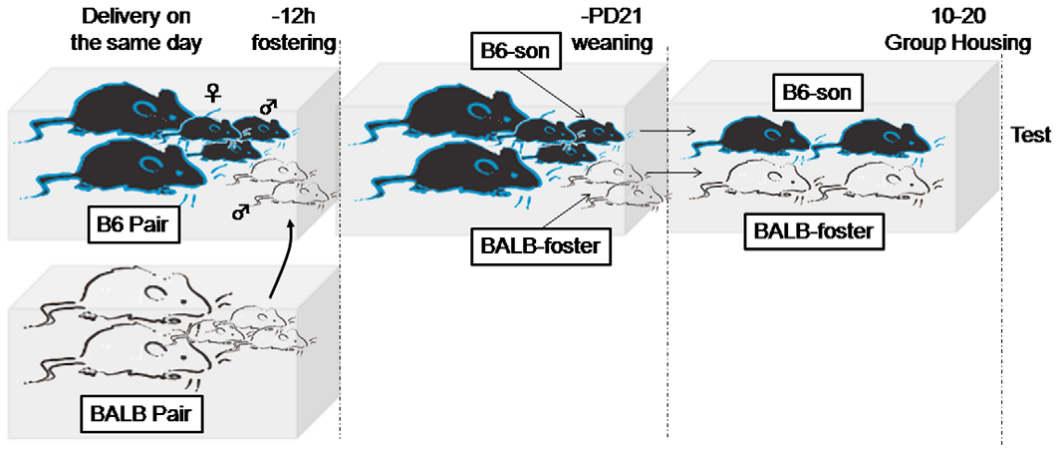
Timeline of the cross-fostering procedure. This figure illustrates the case of cross-fostering from BALB to B6. When newly born pups were found at the same time in both strains of parents, a part of the litter was reciprocally cross-fostered to parents of the other strain of mice. The control mice were handled in the same manner as fostered pups but returned to their own parents. All litters were left undisturbed until weaning (PD21). After weaning,they were housed with males of the non-cross fostered controls of the different strain until ultrasound recording at 10–20 weeks of age.

A male and a female mouse of the same strain were pair-housed in a cage (17.5 cm × 24.5 cm × 12.5 cm) for breeding. When the female was pregnant, delivery was examined every 6–8 hours. When newly born pups were found at the same time in both strains of parents, a part of the litter was reciprocally cross-fostered to parents of the other strain of mice (B6-foster and BALB-foster). The control mice were handled in the same manner as fostered pups but returned to their own parents (B6-son and BALB-son). All litters were left undisturbed until weaning (postnatal day (PD) 21). After PD21, they were housed with males of the non-cross fostered controls of the different strain until ultrasound recording at 10–20 weeks of age (Fig.7). The number of animals and litters (animals/litters) used in this experiment were as follows: B6 (6), BALB (7), B6-son (5/4), B6-foster (5/3), BALB-son (5/4), and BALB-foster (6/5). Because it is known that mating can affect the vocal morphology of male songs, we separately analyzed strain differences between B6 and BALB mice in sexually experienced males and strain and environmental effects between cross-fostered and naturally reared B6 and BALB mice in sexually inexperienced males.

### Ultrasound recording

All experiments were carried out in a soundproof chamber (Muromachi Kikai, Tokyo, Japan) under a red dim light, from 1300 to 1700 hours. Ultrasonic sounds were detected using a condenser microphone (UltraSoundGate CM16/CMPA, Avisoft Bioacoustics, Berlin, Germany) designed for recordings between 10 and 200 kHz. The microphone was connected to an A/D converter (UltraSoundGate 116, Avisoft Bioacoustics, Berlin, Germany) with a sampling rate of 300 kHz and acoustic signals were transmitted to a sound analysis system (SASLab Pro, Avisoft Bioacoustics, Berlin, Germany). During the recording, a subject male mouse was individually housed in a test cage (12.5 cm × 20.0 cm × 11.0 cm) and kept there for at least 2 h for habituation. The test cage was placed in the soundproof chamber, and a female mouse, devocalized by unilateral sectioning of the inferior laryngeal nerve [23], was introduced into the test cage. The ultrasound was recorded for 3 min, and the data were later analyzed.

### Ultrasound analysis

Spectrograms were generated with an FFT-length of 1024 points and a time-window overlap of 75% (100% frame, Hamming window). The spectrogram was produced at a frequency resolution of 488 Hz and a time resolution of 1 ms. A lower cut-off frequency of 20 kHz was used to reduce background noise outside the relevant frequency band. Parameters analyzed for each subject included the number of syllables, duration of syllables, and qualitative and quantitative analyses of sound frequencies measured in terms of frequency at the maximum of the spectrum.

Waveform patterns of calls collected from every group (B6, 6179 syllables; BALB, 6244 syllables; B6-son, 5487 syllables; B6-foster, 6414 syllables; BALB-son, 4973 syllables; BALB-foster, 6963 syllables) were analyzed in detail. Each syllable was identified as 1 of 10 distinct categories, based on internal pitch change, length, and shape, according to previously reported categories with minor modifications (Fig. S1) [14]. The classification of the 10 categories of ultrasonic vocalization syllables is described in the Results section. The frequency of appearance of each category was compared between the groups. In order to confirm the categorization, a likelihood ratio test examining whether there was a systematic difference between the 2 blind experimenters was performed by a generalized linear model that consisted of an explanatory variable (number of syllables) and 3 response variables (2 operators, 11 categories of syllables, and 6 mice). No significant difference was found between the 2 operators (quasi-Poisson error, log link, total number of all syllables in each mouse as offset, *F*_(1,115)_ = 0.13, *p* = 0.72). The occurrence of each syllable was compared between groups using MANOVA, followed by a Bonferonni post-hoc test.

### Sequential analysis of syllables

The prevalence of a syllable type was defined as follows on the basis of a previous study [14]: the syllable types with jumps (1 jump, more jumps) were denoted as A, with all other syllable types denoted as B, and the gap (more than 0.25 s) was Z. One-to-one transition probabilities between these 3 categories were analyzed and indicated by diagrams (Eureka version 1.0 http://sites.google.com/site/eurekawiki/). The occurrence of each transition type was compared between groups using MANOVA, followed by a Bonferonni post-hoc test.

## Supporting Information

Figure S1 **Song syllable characteristics.** Ten categories were defined as follows. Upward: duration of 5–50 ms, frequency increaseof more than 5 kHz from starting point to end. Downward: duration of 5–50 ms, frequency decrease of more than 5 kHz from starting point to end. Flat: duration of 5–35 ms, frequency difference of less than 5 kHz between starting point and end. Short: duration of less than 5 ms, frequency difference of less than 5 kHz between starting point and end. Chevron: duration of 15–80 ms, frequency increase of more than 5 kHz from starting point to frequency peak and frequency increase or decrease of more than 5 kHz from frequency peak to end (*; frequency peak). Wave: duration of 15–100 ms, frequency increase or decrease of more than 5 kHz from starting point to the first frequency peak (or bottom) and containing 1 frequency peak and 1 frequency bottom (*; frequency peak and bottom). Complex: duration of 30–150 ms, frequency increase or decrease of more than 5 kHz from starting point to the first frequency peak (or bottom) and containing more than 3 frequency peaks and/or frequency bottoms that differ from each other by more than 5 kHz in frequency (*; frequency peak and bottom). One jump: duration of 10–50 ms and containing 1 frequency gap (#; frequency gap, less than 1 ms and more than 5 kHz frequency difference). More jumps: duration of 15–100 ms and containing more than 2 frequency gaps (#; frequency gap). Harmonics: duration of 10–100 ms and containing more than 2 Chevron, Wave, Complex, One jump, or More jumps syllables in parallel with a main syllable that has the highest dB count.
(TIF)

Figure S2 **Sequential analysis of syllable types in B6 and BALB mice.** The sequential analyses of 10 categories of syllables demonstrated a very complicated transition both in B6 (upper) and BALB (lower) mice. a: upward, b: downward, c: flat, d: short, e: chevron, f: wave, g: complex, h: one jump, i: more jumps, j: harmonics, Z: gap.
(TIF)

Audio S1 **B6 male song.**
(WAV)

Audio S2 **BALB male song.**
(WAV)

Audio S3 **B6-son male song.**
(WAV)

Audio S4 **B6-foster male song.**
(WAV)

Audio S5 **BALB-son male song.**
(WAV)

Audio S6 **BALB-foster male song.**
(WAV)
