# Supplementary figures and images for: Cross Fostering Experiments Suggest That Mice Songs Are Innate
Source: PLoS One. 2011 Mar 9;6(3):e17721. doi: 10.1371/journal.pone.0017721 (PMC3052373; doi:10.1371/journal.pone.0017721)

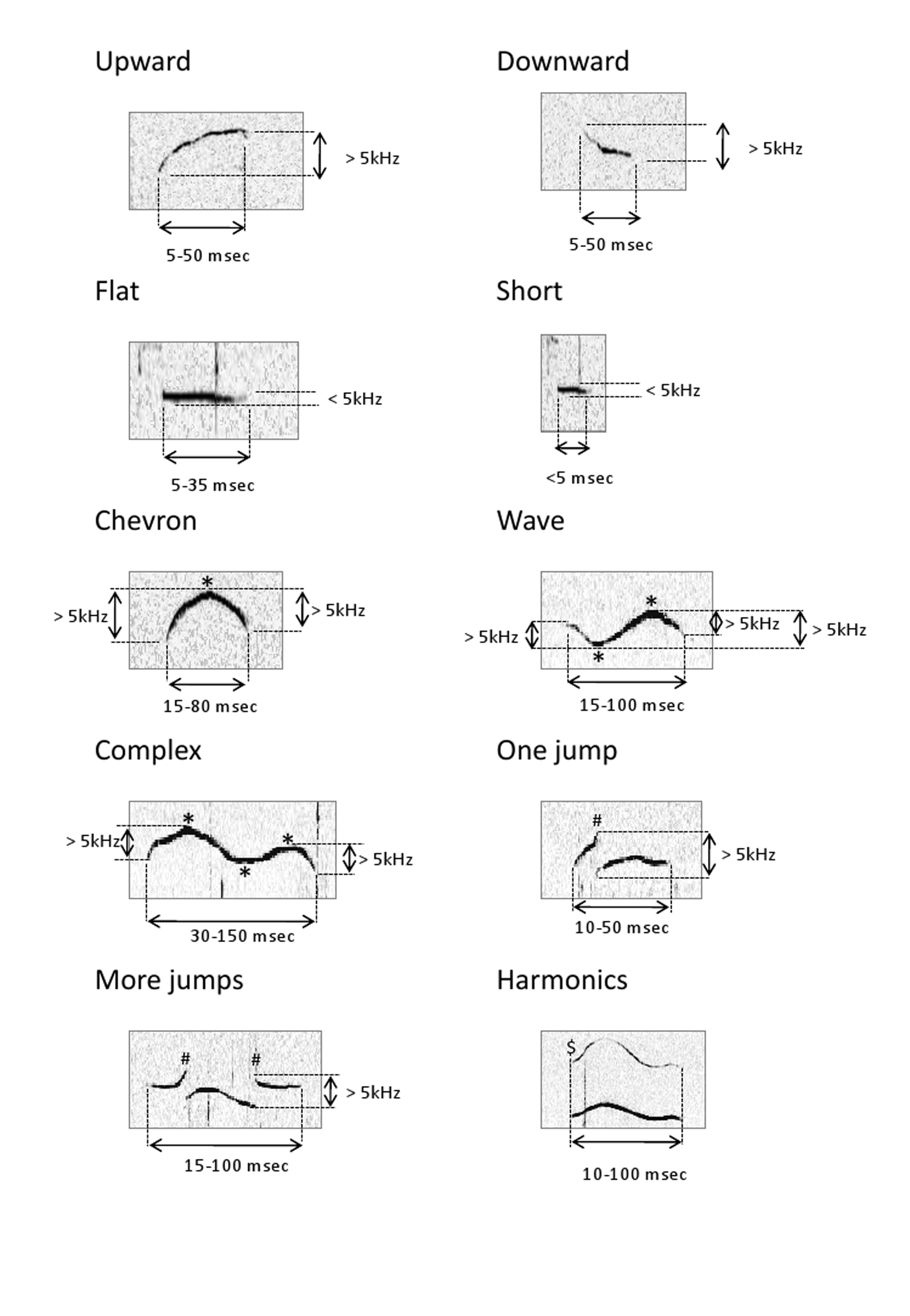

Supplement: Figure S1 — Song syllable characteristics. Ten categories were defined as follows. Upward: duration of 5–50 ms, frequency increaseof more than 5 kHz from starting point to end. Downward: duration of 5–50 ms, frequency decrease of more than 5 kHz from starting point to end. Flat: duration of 5–35 ms, frequency difference of less than 5 kHz between starting point and end. Short: duration of less than 5 ms, frequency difference of less than 5 kHz between starting point and end. Chevron: duration of 15–80 ms, frequency increase of more than 5 kHz from starting point to frequency peak and frequency increase or decrease of more than 5 kHz from frequency peak to end (*; frequency peak). Wave: duration of 15–100 ms, frequency increase or decrease of more than 5 kHz from starting point to the first frequency peak (or bottom) and containing 1 frequency peak and 1 frequency bottom (*; frequency peak and bottom). Complex: duration of 30–150 ms, frequency increase or decrease of more than 5 kHz from starting point to the first frequency peak (or bottom) and containing more than 3 frequency peaks and/or frequency bottoms that differ from each other by more than 5 kHz in frequency (*; frequency peak and bottom). One jump: duration of 10–50 ms and containing 1 frequency gap (#; frequency gap, less than 1 ms and more than 5 kHz frequency difference). More jumps: duration of 15–100 ms and containing more than 2 frequency gaps (#; frequency gap). Harmonics: duration of 10–100 ms and containing more than 2 Chevron, Wave, Complex, One jump, or More jumps syllables in parallel with a main syllable that has the highest dB count. (TIF) [file pone.0017721.s001.tif]

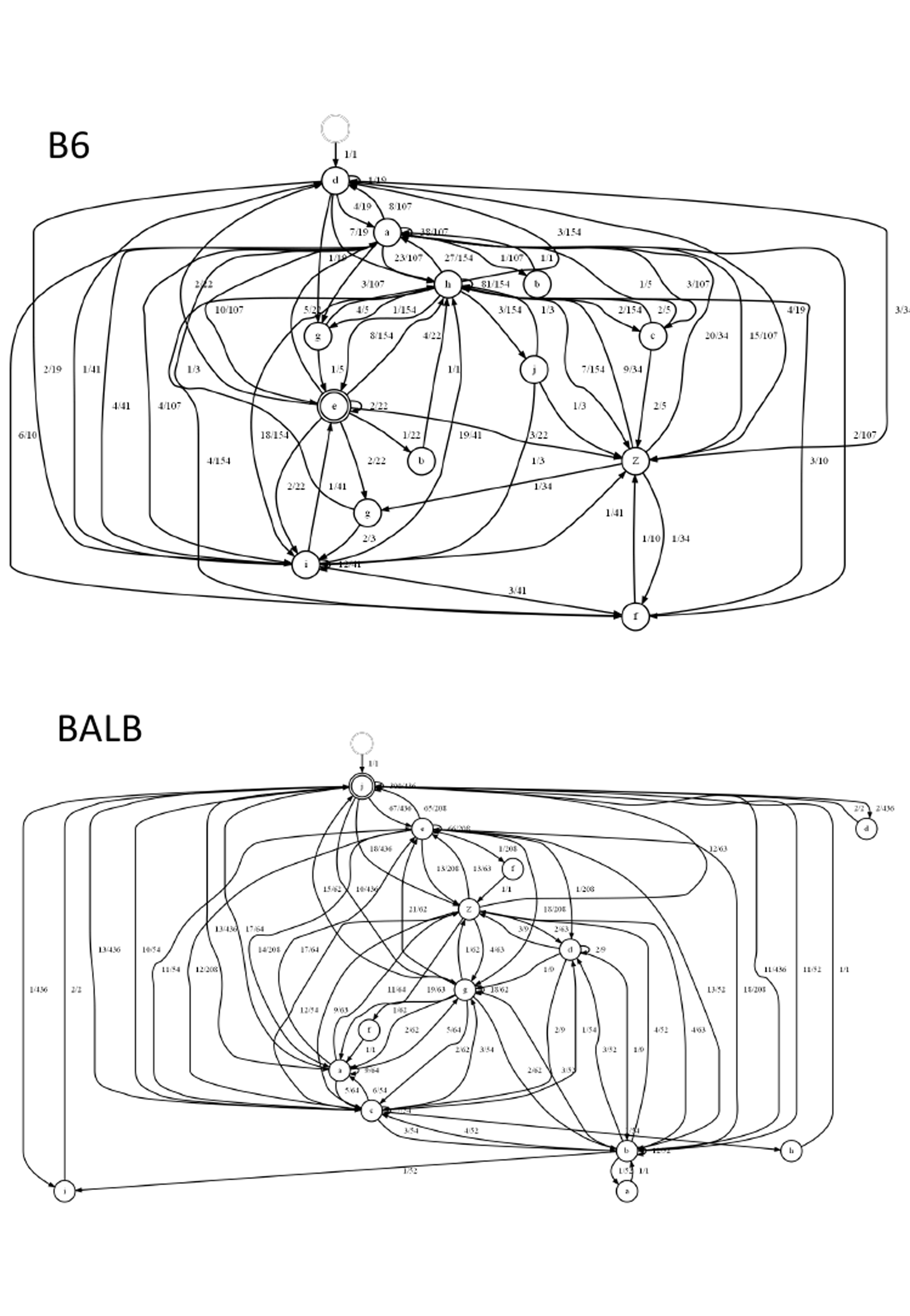

Supplement: Figure S2 — Sequential analysis of syllable types in B6 and BALB mice. The sequential analyses of 10 categories of syllables demonstrated a very complicated transition both in B6 (upper) and BALB (lower) mice. a: upward, b: downward, c: flat, d: short, e: chevron, f: wave, g: complex, h: one jump, i: more jumps, j: harmonics, Z: gap. (TIF) [file pone.0017721.s002.tif]
